# Defective regulation of POMC precedes hypothalamic inflammation in diet-induced obesity

**DOI:** 10.1038/srep29290

**Published:** 2016-07-04

**Authors:** Gabriela F. P. Souza, Carina Solon, Lucas F. Nascimento, Jose C. De-Lima-Junior, Guilherme Nogueira, Rodrigo Moura, Guilherme Z. Rocha, Milena Fioravante, Vanessa Bobbo, Joseane Morari, Daniela Razolli, Eliana P. Araujo, Licio A. Velloso

**Affiliations:** 1 Laboratory of Cell Signaling, University of Campinas, 13084-970 – Campinas-SP, Brazil; 2 Department of Internal Medicine, University of Campinas, 13084-970 – Campinas-SP, Brazil; 3 Faculty of Nursing, University of Campinas, 13084-970 – Campinas-SP, Brazil

## Abstract

Obesity is the result of a long-term positive energy balance in which caloric intake overrides energy expenditure. This anabolic state results from the defective activity of hypothalamic neurons involved in the sensing and response to adiposity. However, it is currently unknown what the earliest obesity-linked hypothalamic defect is and how it orchestrates the energy imbalance present in obesity. Using an outbred model of diet-induced obesity we show that defective regulation of hypothalamic POMC is the earliest marker distinguishing obesity-prone from obesity-resistant mice. The early inhibition of hypothalamic POMC was sufficient to transform obesity-resistant in obesity-prone mice. In addition, the post-prandial change in the blood level of β-endorphin, a POMC-derived peptide, correlates with body mass gain in rodents and humans. Taken together, these results suggest that defective regulation of POMC expression, which leads to a change of β-endorphin levels, is the earliest hypothalamic defect leading to obesity.

Increased consumption of dietary fats is one of the leading causes of obesity, which affects, currently, more than 500 million people around the world^1^,^2^. Recent studies have shown that dietary fats promote obesity not only because of their caloric value but also because they can damage the hypothalamic neuronal circuitries that control whole body energy homeostasis^3^,^4^,^5^,^6^. An interesting epidemiological aspect of the widespread rise in the prevalence of obesity is that even with an almost unlimited access to energy-dense foods, the disease is expected to affect no more than 50% of the exposed humans^7^. In fact, recent studies have reported a slow-down in the rate of obesity prevalence growth in some regions^8^,^9^. At least part of this protection is likely due to genetic factors, and identifying mechanisms linked to this protective genotype may unveil novel potential targets for the treatment of obesity.

Similarly to human populations, outbred strains of rodents can also present some degree of protection against obesity when exposed to unlimited amounts of dietary fats^10^. Swiss mice belong to an outbred strain related to the diabetes-prone AKR^11^,^12^. When exposed to unlimited amounts of dietary fat, approximately 50% of the animals become obese and diabetic^12^,^13^. In the present study we developed a strategy to identify the 25% of animals with the highest predisposition to or highest protection against obesity. We hypothesized that differences in the predisposition to obesity could result from an anomalous activity of hypothalamic neurons. The selected mice were employed in experiments aimed at identifying the earliest hypothalamic functional changes related to diet-induced obesity. We show that the anomalous regulation of hypothalamic POMC precedes inflammation and is a determining factor leading to the progression of obesity. Moreover, in rodents and humans, blood levels of β-endorphin, a subproduct of the POMC transcript, is regulated differently in obesity-prone and obesity-resistant subjects.

## Results

### Hypothalamic inflammation and anomalous regulation of POMC/NPY are early events that distinguish obesity-prone from obesity-resistant mice

**Figure 1:**
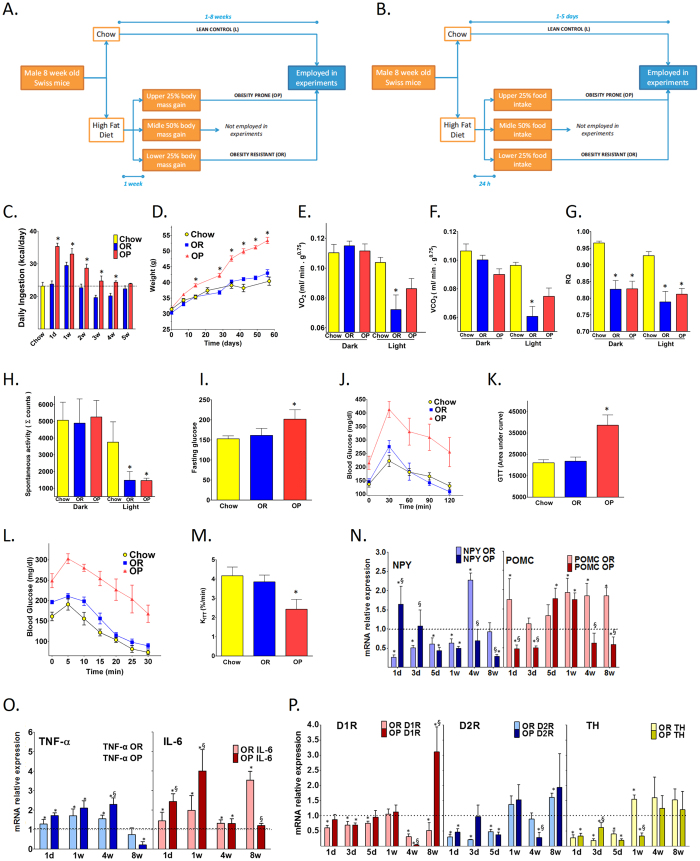
Characterization of the obese prone and obese resistant mice. (**A,B**) are schematic representations of the protocols used in most experiments of this study for selecting obese-prone (OP) and obese-resistant (OR) mice. Upon selection, OP and OR mice, and chow fed controls were evaluated for caloric intake (**C**) body mass gain (**D**) O_2_ consumption (**E**) CO_2_ production (**F**) respiratory quotient (RQ) (**G**) spontaneous activity (**H**) fasting glucose levels (**I**) blood glucose (**J**) and area under the glucose curve (**K**) during an oral glucose tolerance test, blood glucose (**L**) and constant of glucose decay (Kitt) (**M**) during an insulin tolerance test. NPY/POMC (**N**) TNF*α*/IL6 (**O**) and D1R/D2R/TH (**P**) transcript levels were measured in the hypothalamus of OP and OR mice fed on a high-fat diet (HFD) for 1 day to 8 weeks. In C, the dashed line indicates the daily ingestion of control mice fed on chow. In N-P, the results are presented as relative to the level of respective transcripts expressed in the hypothalamus of mice fed on chow (dashed line). In all experiments n = 6; *p < 0.05 *vs.* chow; ^§^p < 0.05 *vs.* respective OR.

Two distinct protocols were employed to screen Swiss mice for the predisposition or resistance to obesity. In the first protocol (Fig. 1A), Swiss mice were fed on an HFD for one week and body mass gain was recorded. Mice on the upper quartile were selected as obesity-prone (OP), while mice on the lower quartile were selected as obesity-resistant (OR) (Fig. 1A). A similar result in identifying the distinct phenotypes could be achieved employing a one-day protocol (Fig. 1B) and determining food intake as an indication of obesity resistance or predisposition. Thus, highest caloric intake resulted in the OP phenotype while lowest caloric intake resulted in the OR phenotype (Fig. 1B). There was a high correlation between both these protocols (r = 0.8; p < 0.05) and, therefore, they were employed in most of the experiments of this work. OP mice are hyperphagic during the first four weeks (Fig. 1C), are heavier than OR as early as seven days after HFD introduction, and 60 days later the OP mice present a 1.5-fold higher body mass than OR and lean controls (Fig. 1D). O_2_ consumption/CO_2_ production, respiratory quotient (RQ) and spontaneous activity are roughly similar between OP and OR mice and apart from chow control mice (Fig. 1E–H) and should not be responsible for the weight gain pattern observed. Together, these results suggest that distinction in body mass between the groups is a result of increased caloric intake during the first four weeks. As expected, OP are hyperglycemic (Fig. 1I), glucose intolerant (Fig. 1J,K) and insulin resistant (Fig. 1L,M) as compared to OR and chow control mice.

We hypothesized that the distinct regulation of brain/hypothalamic neuropeptides involved in the control of feeding or the activation of hypothalamic inflammation would be very early events involved in the phenotypic distinction between OP and OR mice. To test this hypothesis we performed a time-course evaluation of the expressions of POMC/NPY system markers, the dopaminergic system and inflammation in the hypothalami of OP and OR mice. As depicted in Fig. 1N, as early as one day after the introduction of an HFD, both NPY and POMC are anomalously regulated in the hypothalamus of OP mice, presenting an unexpected increase in NPY and a reduction of POMC as compared to OR and chow mice. Only after five days and one week, the expressions of NPY and POMC, respectively, become similar between OP and OR, reaching levels that would be predicted for mice fed on an HFD, i.e., reduced NPY and increased POMC as compared to chow controls. However, after four and eight weeks the expressions of both NPY and POMC are, again, distinct between OP and OR, with a reduced expression of both neuropeptides in OP mice. The expressions of TNF*α* and IL6 are increased in the hypothalami of OP and OR mice throughout most of the experimental period; however, OP mice present a more remarkable increase of IL6, which is significantly higher than OR at days one and seven, and OP TNF*α* levels are higher than OR and chow four weeks after the introduction of HFD (Fig. 1O). Figure 1P shows that transcripts of the dopaminergic system; D1R and D2R, receptors for dopamine, and tyrosine hydroxylase (TH), enzyme involved in dopamine synthesis, are in general similarly regulated in OP and OR throughout most of the experimental period. There are only a few conditions in which the expression of transcripts differs between OR and OP: at three days, TH is higher in OP; at one week, TH is lower in OP; at four weeks, DR1 and DR2 are lower in OP; and, at eight weeks, DR1 is higher in OP.

### Defective regulation of POMC is the earliest event distinguishing OP from OR mice, and hypothalamic and blood β-endorphin levels are differentially regulated in response to caloric intake in OP and OR rodents

**Figure 2:**
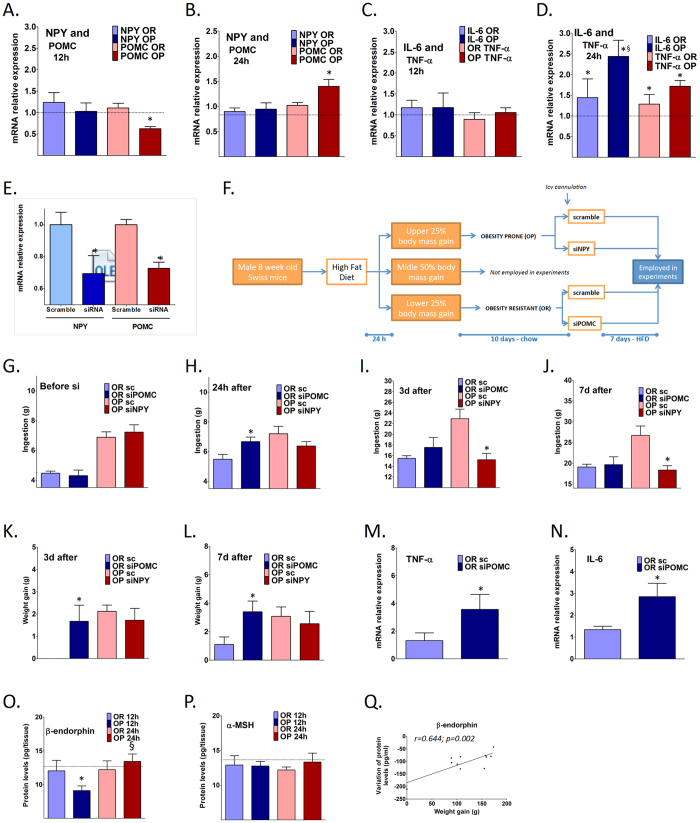
Anomalous regulation of hypothalamic POMC is the earliest event distinguishing OP from OR mice. NPY/POMC (**A,B**) and IL6/TNF*α* (**C,D**) transcripts levels were measured in the hypothalamus of obese-prone (OP) and obese-resistant (OR) mice fed on a high-fat diet for 12 and 24 h, respectively, and samples were collected without fasting. Panel E depicts the real-time PCR determination of NPY and POMC transcript expression in the hypothalamus of mice treated either with NPY or POMC small-interfering RNAs (siRNA); control expression was obtained by the treatment with scramble siRNA. Panel F depicts the protocol employed for experiments presented in panels G–N. For that, OP mice were treated either with a scramble or an anti-NPY small interfering RNA (sc or siNPY, respectively) and OR mice were treated either with a scramble or an anti-POMC small interfering RNA (sc or siPOMC, respectively); food intake was determined before (**G**) and 24 h (**H**) three days (**I**) and seven days (**J**) after siRNA treatment; NPY/POMC transcripts were determined in the hypothalamus three (**K**) or seven (**L**) days after siRNA treatment; TNF*α* (**M**) and IL6 (**N**) transcripts levels were measured in the hypothalamus of OR mice treated either with a scramble or an anti-POMC small interfering RNA (sc or siPOMC, respectively). In (**O,P**) β-endorphin and *α*-MSH, respectively, were determined using ELISA in hypothalamic protein extracts obtained 12 or 24 h after introduction of a high-fat diet for OP and OR mice. In (**Q**) the variations of rat blood β-endorphin levels, determined using ELISA, from base-line to 12 h after introduction of a high-fat diet were plotted against body mass gain during a four-week period feeding on a high-fat diet. In (**A–D**) the results are presented as relative to the level of respective transcripts expressed in the hypothalamus of mice fed on chow (dashed line). In E, the results are presented as transcript expression relative to scramble treated controls. In (**O,P**) dashed line indicates values of mice fed on chow. In all experiments n = 6; in (**A–D**) *p < 0.05 *vs.* chow; ^§^p < 0.05 *vs.* respective OR; in (**E**) *p < 0.05 vs. respective scramble treated controls; in (**G–N**) *p < 0.05 *vs.* respective sc; in (**O–P**) *p < 0.05 *vs.* chow; ^§^p < 0.05 *vs.* respective 12 h.

Since NPY/POMC and IL6 transcripts were differently regulated between OP and OR as early as 24 h after the introduction of HFD, we decided to measure these markers after a shorter period of exposure to HFD. In the experiments shown in Fig. 1N,O, mice were fed on HFD for 24 h and samples were collected after a 12 h fast. When mice are fed for 12 h (Fig. 2A) or 24 h (Fig. 2B) on an HFD and samples are collected without fasting, only POMC is differentially regulated between OP and OR; thus, 12 h on an HFD leads to a reduction of POMC and 24 h on an HFD leads to an increase of POMC in OP mice. In addition, we measured transcripts of markers of inflammation. As depicted in Fig. 2C, neither IL6 nor TNF*α* were modulated after 12 h on an HFD, whereas in OP mice, IL6 was significantly increased after 24 h  h on an HFD (Fig. 2D). These findings suggest that NPY/POMC imbalance is the early event that distinguishes OP from OR mice, most likely through the POMC pathway, followed shortly thereafter by the inflammatory pathway.

Given that OR mice fed on HFD express high levels of POMC, whereas OP mice fed on HFD express high levels of NPY in the hypothalamus (Fig. 1N), we hypothesized that inhibiting POMC in OR and NPY in OP mice could revert their phenotypes. In order to explore this hypothesis, OR mice were treated with a siRNA against POMC (which resulted in a 30% inhibition of hypothalamic POMC, Fig. 2E), while OP mice were treated with a siRNA against NPY (which resulted in a 35% inhibition of hypothalamic NPY, Fig. 2E), as depicted in the illustrative protocol in Fig. 2F. Additionally, OP mice were treated with a siRNA against POMC and OR mice were treated with a siRNA against NPY. As these approaches resulted in no phenotypic change they are presented as supplementary material (Supplementary Figures 1 and 2). Figure 2G–J show that inhibition of POMC in OR results in the earliest alteration in caloric intake, increasing HFD consumption after 24 h. On the other hand, inhibition of NPY in OP results in reduced caloric intake after three and seven days on an HFD; however, this was accompanied by only a trend for body mass reduction. Despite the fact that inhibition of NPY in OP resulted in an apparently more robust effect on caloric intake as compared to the inhibition of POMC in OR, only the inhibition of POMC in OR produced a modification of the obese phenotype, as depicted in Fig. 2K,L; thereby, upon POMC inhibition, OR body mass gain becomes similar to OP body mass gain. In addition, the inhibition of POMC in OR mice resulted in increased hypothalamic expression of the inflammatory cytokines TNF*α* (Fig. 2M) and IL6 (Fig. 2N). These results corroborate our hypothesis that POMC defect is the earliest event involved in OP versus OR differentiation, followed by inflammation activation.

Given that POMC transcript is processed to β-endorphin and *α*-MSH, the question arises as to whether there is a POMC-derived product more involved in OP versus OR phenotype development. It must also be highlighted that OP POMC levels dropped and raised after 12 and 24 h of HFD, respectively, while OR mice kept the POMC levels at the same levels as the chow control mice (Fig 2A). So, hypothesizing that the magnitude of POMC-derived peptide levels changes in concert with a propensity to gain body mass, we evaluated the variation in the hypothalamic levels of β-endorphin and *α*-MSH in OP and OR mice after 12 and 24 h of HFD exposition. As depicted in Fig. 2O, there is a marked difference in hypothalamic β-endorphin variation between 12 h and 24 h after HFD introduction for OP and OR mice, on the other hand there were no significant changes in *α*-MSH levels (Fig. 2P).

Because β-endorphin can be measured in the blood and is correlated to hypothalamic levels^14^, we tested the relationship between the variation of blood β-endorphin levels before and 12 h after HFD consumption versus four-week weight gain in OP and OR rats and found that indeed there was a significant correlation between these two variables (Fig. 2Q): the higher the variance of β-endorphin levels, the higher the weight gain after four weeks of an HFD.

### Changes in blood β-endorphin levels are correlated with body mass gain in lean male humans

**Figure 3:**
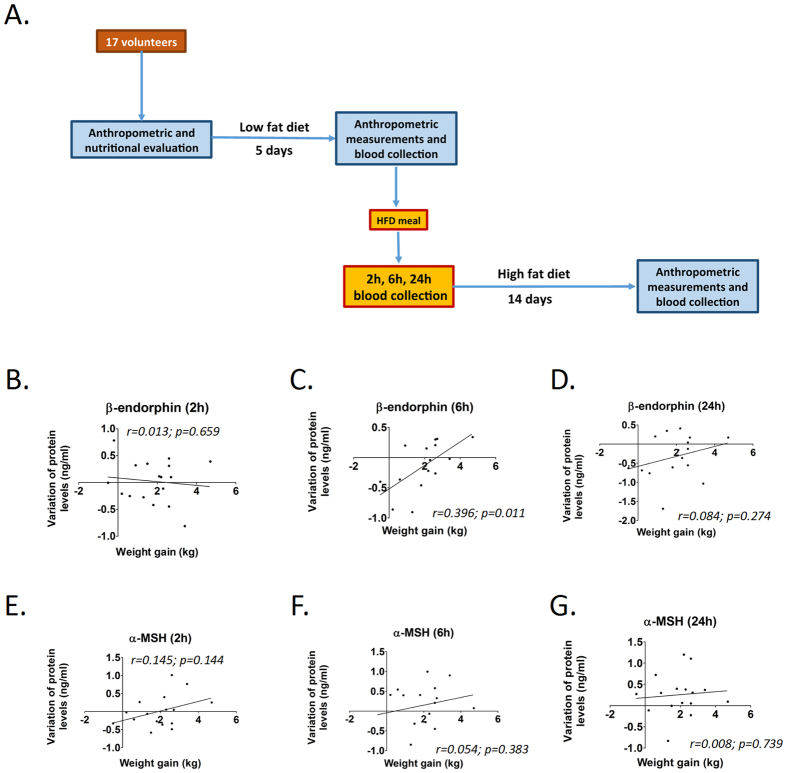
Positive correlation between blood β-endorphin variation and body mass gain in humans. Panel A illustrates the protocol employed to evaluate variation of blood β-endorphin and *α*-MSH in humans. Changes in blood β-endorphin (**B–D**) and *α*-MSH (**E–G**) levels between base-line and 2 h (**B**,**E**), 6 h (**C,F**) and 24 h after a high fat meal (**D,G**) were plotted against 15 days body mass gain in humans on a high-fat diet. In all experiments n = 17.

Seventeen volunteers were recruited and submitted to a protocol to evaluate the blood levels of β-endorphin and *α*-MSH before and after a high fat meal, as depicted in Fig. 3A. Anthropometric data of subjects is presented in Supplementary Table 1. We compared the changes in blood levels of β-endorphin and *α*-MSH between base-line and 2 h, 6 h or 24 h after a high fat meal and the variation of body mass after 14 days on an HFD (Fig. 3B–G). As depicted in Fig. 3C, there was a significant positive correlation between the variation of blood levels of β-endorphin from base-line to 6 h and the increase of body mass during a 14 d period on an HFD. On the other hand, there were no significant differences for the variation of β-endorphin in the other intervals or *α*-MSH in any interval tested.

## Discussion

Defining the earliest pathophysiological events that determine the onset of obesity may contribute to the development of strategies aimed at preventing this highly prevalent disease. The hypothalamus has an important role in the regulation of whole body energy stores and early defects in its function may impact on the genesis of obesity^15^,^16^. First-order neurons of the arcuate nucleus expressing NPY/AgRP or POMC/CART can sense peripheral signals of adiposity delivered by leptin and insulin, as well as signals of satiety delivered by a combination of several gut hormones, such as ghrelin, GLP1, CCK, among many others^17^. In response to such signals, the hypothalamus coordinates a neuronal network that controls caloric intake, thermogenesis, spontaneous physical activity and distinct aspects of whole body metabolism^15^. Because of their central role in energy homeostasis, NPY/AgRP and POMC/CART neurons are a focus of intense investigation aimed at defining defects involved in the development of obesity as well as in the search for therapeutic strategies to revert the obese phenotype^18^.

The importance of the melanocortin system in obesity is illustrated by the fact that mutations of MC4R are the most common causes of monogenic obesity, leading to a phenotype of extreme adiposity^19^. However, in non-monogenic obesity, genome-wide association studies (GWAS) showed a relatively weak association between adiposity and genetic regions related to the melanocortin system, particularly the MC4R gene^20^,^21^,^22^. Therefore, it is possible that in most cases of obesity, the activity of hypothalamic neurons is modulated by a combination of polygenic traits allied to external factors.

Components of the diet are amongst the most important factors that modulate hypothalamic function. Studies have shown that dietary fats can promote severe damage in neurons of the medium-basal hypothalamus^3^,^4^,^5^,^23^,^24^,^25^. The local inflammation triggered by dietary fats can, early on, produce hypothalamic resistance to the catabolic actions of leptin and insulin^3^,^26^,^27^. Upon prolonged exposure to dietary fats, neurons, particularly POMC, can undergo apoptosis^23^.

The dopaminergic reward system is also capable of modulating the activity of medium-basal hypothalamic neurons^28^. Studies have shown that the choice for palatable foods, some of them rich in dietary fats, results from a complex interaction between the mesolimbic dopaminergic system and neurons of the hypothalamus^29^. Moreover, a number of medications used to treat obesity act through dopaminergic receptors in the hypothalamus, providing a pharmacological basis for the connection between POMC and NPY neurons and the dopaminergic system^30^.

In the present study, we evaluated the impact of dietary fats on the regulation of these three systems/mechanisms potentially involved in the development of obesity. Our strategy was to compare OP and OR mice from an outbred strain. It has been previously shown that in outbred strains, obesity develops as a consequence of a combination of polygenic and environmental factors, just as in human populations^31^. Our findings corroborate and extend previous studies showing that mice develop obesity mostly because of increased caloric intake during the first four weeks on an HFD^31^,^32^. In addition to increased body mass, OP mice became diabetic and insulin-resistant as previously reported^33^.We performed a time-course evaluation of the hypothalamic expressions of transcripts encoding proteins that belong to the three systems evaluated in this study: i, NPY/POMC; ii, dopaminergic system; and, iii, inflammation. There were only three out of seven transcripts that presented different expressions between OP and OR during the first three days on an HFD: NPY, POMC and IL6. NPY and IL6 were higher, while POMC was lower in OP as compared to OR.

A number of studies have evaluated the hypothalamic expression of neurotransmitters and components of the dopamine reward system in OP and OR mice. Most of the studies evaluated mice either before^34^ or medium/long term^32^,^35^ after the introduction of dietary fats. In outbred rats, NPY hypothalamic expression is increased in OP before the introduction of dietary fat and independently of fasting^34^. In the long run, if mice are maintained on chow, NPY remains higher in OP than OR^32^. However, upon consumption of an HFD, NPY levels are reduced both in OP and OR^32^, which is quite similar to our findings when mice were fed on an HFD for eight weeks. Another study showed that OP mice present low levels of POMC when chronically fed on an HFD, which is accompanied by a similar reduction of AgRP, which is co-expressed with NPY^35^. Regarding the dopaminergic system, it has been previously shown that long-term feeding on an HFD tends to promote an increase in the hypothalamic expression of most of the components of the system, both in OP and OR^36^. However, the increase is more important in OP than OR^36^, a result that matches our findings when mice were fed on an HFD for eight weeks. As an attempt to define the earliest hypothalamic event that segregates OP from OR mice we employed a 12 h HFD protocol to separate OP and OR mice and only POMC was different between OP and OR 12 h after introduction of the diet, preceding changes in NPY and IL6. These data suggest that POMC/NPY controls the amount of calories consumed and, since composition and amount of diet consumed are important factors controlling hypothalamic inflammation, one can conclude that POMC/NPY controls, indirectly, the inflammatory pathway. As shown in Fig. 1, OP mice became obese mostly because of increased caloric intake during the first four weeks of experiment. During most of this time the levels of hypothalamic POMC were lower in OP than OR mice. Moreover, when we inhibited POMC in the hypothalamus of OR mice, we were capable to transforming OR in OP mice, which was accompanied by increased diet-induced inflammation of the hypothalamus. Importantly, neither the inhibition of NPY nor the inhibition of POMC in OP mice were capable of modifying their phenotype, which reinforces the hypothesis that an early reduction of POMC following the introduction of a HFD is involved in the increased predisposition to obesity. Regarding the impact of manipulating hypothalamic POMC on the development of obesity, a study has shown that in mice with conditional expression, the early switch-off of hypothalamic POMC is accompanied by the development of obesity, which can be completely rescued only if POMC is reactivated before the fourth week of life^37^.

It has been shown that the regulation of food intake by the melanocortin system depends on the preferential POMC product that is expressed after transcript processing. The activation of the cannabinoid receptor, CBR1, which is known to mediate cannabinoid-induced food intake, leads to the increased neuronal activity of POMC neurons and the preferential expression of β-endorphin instead of *α*-MSH^38^. In the final part of the study we evaluated the expression of β-endorphin and *α*-MSH in the hypothalamus and blood of rodents. We found that hypothalamic β-endorphin levels change through the first hours of HFD exposition in OP mice and there is a significant direct correlation between blood β-endorphin variation after HFD introduction and body mass change; on the other hand, this was not observed for *α*-MSH. Not only does this relation between variation in β-endorphin levels and hyperphagia offer one mechanistic explanation for the higher weight gain in OP rodents, but it also provides a potential marker to obesity propensity. Therefore, we performed a short-term evaluation of the impact of dietary fat consumption by a group of lean human volunteers. We found that, similarly to rodents, the acute change in human blood β-endorphin in response to dietary fat was correlated with body mass gain during a period of overeating. In other words, both in rodents and humans, a larger early decrease in β-endorphin levels after the consumption of a high-fat meal is correlated with less weight gain.

One intriguing question raised as a consequence of the results of this study is that despite the fact that POMC hypothalamic expression is increased in OP mice fed on HFD, as depicted in Fig. 2B, mice are hyperphagic and gain more weight. It would be intuitive to believe that increased POMC levels would be accompanied by reduced caloric intake and less body mass gain. Nevertheless, POMC can be processed into at least two products that impact directly on caloric intake, *α*-MSH and β-endorphin, one reducing and the other increasing caloric intake, respectively. Our results provide evidence to suggest that the abnormal processing of POMC into β-endorphin may play a major role in the early response to a HFD meal impacting on long-term body mass gain.

Studies have shown elevated blood β-endorphin levels in different groups of obese humans^39^. In addition, it was shown that the rise of blood β-endorphin following the ingestion of glucose was significantly higher in obese subjects^40^. However, no previous study has evaluated whether changes of blood β-endorphin following a meal could correlate with body mass gain after a period of overeating. Despite the fact that our results were obtained with a small group of volunteers accompanied during a short period of overeating, they match the results obtained in rodents and indicate that β-endorphin may be an interesting target to determine obesity predisposition.

In conclusion, the anomalous regulation of hypothalamic POMC expression in response to dietary fats is the earliest phenomenon distinguishing OP from OR, and at least one product of the POMC transcript, β-endorphin, undergoes change in the blood in correlation with body mass change after a period of high fat exposition, both in rodents and humans. Based on the results of this study, we propose that the early variation in the levels of POMC following a meal, and preferentially of one of its products, β-endorphin, predicts body mass gain over a short period of overfeeding. Future investigations at clinical level should determine if this variation could be an effective marker to predict whether someone is prone to obesity development.

## Methods

### Experimental animals and protocols

All mice and rats were maintained in individual cages at 23 ± 2 °C, with a 12/12 h dark-light cycle and diet and water *ad libitum*. Experimental animals were fed on chow from weaning until eight weeks of life, and then they were randomly selected for either continuing on chow or transferred to a high-fat diet (HFD). The macronutrient composition of diets was previously published^41^. Body mass and caloric intake were monitored weekly. In experiments aimed at evaluating the blood levels of β-endorphin and *α*-MSH we employed male Wistar rats because the volume of plasma required for the measurements were larger than what could be obtained from mice. Experiments for blood and tissue extractions were always performed between 8 and 10 AM. Experimental animals were obtained from the University of Campinas Animal Breeding Center. All experiments were performed in accordance with the guidelines of the Brazilian College for Animal Experimentation and were approved by the Ethics Committee at the University of Campinas.

### Obesity prone and resistant protocol

Eight-week old male Swiss mice were fed a high-fat diet (HFD) for 24 h up to eight weeks. As shown in Fig. 1A,B, protocols of one week or one day were employed for selecting mice according to their predisposition or resistance to developing obesity. Mice were grouped into quartiles for total caloric ingestion or weight gain, and only the top quartile [obesity-prone (OP)] and the bottom-quartile [obesity-resistant (OR)] mice were employed in the study. Mice fed on chow throughout the experimental period were used as controls.

### Real Time PCR

Hypothalamic total RNA was extracted using TRIzol reagent (Invitrogen), according to the instructions provided by the manufacturer. The cDNA synthesis was performed using 3.0 μg of total RNA according the manufacturer’s instructions (High Capacity cDNA Reverse Transcription Kit, Life Technologies). Normalization was obtained by determining the expression of glyceraldehyde-3-phosphate dehydrogenase in all samples. Each PCR contained 25–40 ng of reverse-transcribed RNA, 2.5 μl of each specific primer, Taqman Universal master mix (4369016, Life Technologies), and RNAse free water to a 10 μl of final volume. Real time PCR analysis of gene expression was carried out in an ABI Prism 7500 sequence detection system (Applied Biosystems). Primers were purchased from Applied Biosystems and were: NPY, Mm00445771_m1; POMC, Mm00435874_m1; D1R, Mm 01353211_m1; D2R, Mm 00438545m1; TH, Mm 00447557_m1; TNF-alpha, Mm 99999068_m1; IL-6, Mm99999064_m1; GAPD 4352339E for mouse.

### Metabolic characterization

Glucose and insulin tolerance tests were performed in six-hour fasting mice as previously described^41^. The energy expenditure and spontaneous activity were performed in open circuit calorimeter system chambers (LE405 Gas Analyzer, Panlab-Harvard Apparatus, Holliston) as described^41^.

### Short interfering RNA(siRNA) experiments

OP and OR mice selected according to the 24 h protocol (Fig. 1B) were returned to chow for a washout period of ten days. Thereafter, OP mice were assigned to either NPY siRNA or control siRNA treatment and OR mice were assigned to either POMC siRNA or control siRNA groups. NPY siRNA (sc-42100), POMC siRNA (sc-37278) and control siRNA (sc-37007) were from Santa Cruz Biotechnology, Inc. The siRNAs (20 pmol) were infused into the dorsal third ventricle. The animals were allowed to recover for one day on a chow diet, and then transferred to an HFD for seven days. Body weight and food intake were monitored daily. At the end of the experimental period, hypothalami were harvested for the determination of neurotransmitter expression.

### Determination of hypothalamic and blood α-MSH and β-endorphin levels

Hypothalamic and blood *α*-MSH and β-endorphin concentrations were assessed by ELISA (MBS723969, MyBioSource Inc. and MBS741689, MyBioSource Inc.) in accordance to instructions provided by the manufacturer. For hypothalamic determinations, protein extracts were obtained 12 or 24 h after introduction of a high-fat diet for OP and OR mice The plasma *α*-MSH and β-endorphin concentrations were assessed by ELISA (MBS720312, MyBioSource Inc. and MBS733830, MyBioSource Inc.), in rats and humans, in accordance to instructions provided by the manufacturer. In rats, blood samples were collected at base-line and 12 h after the introduction of a high-fat diet. In humans, blood samples were collected at base-line and 2, 6 and 24 h after the ingestion of a high-fat breakfast.

### Human studies

Seventeen healthy male volunteers were included in the study. All subjects gave their informed consent to the study, which was approved by the University of Campinas Ethics Committee. All experimental procedures were carried out in accordance to the National Institutes of Health Regulations, Policies and Guidance for human research. The criteria for inclusion of patients were: i, men between 18 and 25 years of age; ii, body mass index (BMI) between 17 and 25. The exclusion criteria were: i, be diagnosed with diabetes mellitus; ii, be diagnosed with any inflammatory, neoplastic or infectious disease; iii, be diagnosed with hypertension; iv, refer use of psychotropic or anti-inflammatory drugs; v, refer use of substance of abuse or addiction. Upon inclusion, subjects were evaluated by a nutritionist and instructed to consume, *ad libitum*, a low-fat diet (Supplementary Table 2) for five days. On the sixth day, following an overnight fast, blood was collected and subjects were submitted to anthropometric measurements and dual-energy X-ray absorptiometry (DEXA) scan. Thereafter, subjects were offered a high-fat breakfast (Supplementary Table 3), and blood samples were taken 2 h, 6 h and 24 h after the beginning of the breakfast. For the next 14 days, subjects were instructed to consume a high-fat diet (Supplementary Table 4). At the end of the experimental period, subjects were once more submitted to anthropometric measurements and dual-energy X-ray absorptiometry (DEXA) scan. Blood levels of *α*-MSH and β-endorphin were determined by ELISA (EK-043-01CE, Phoenix Pharmaceuticals Inc. and EK-022-14, Phoenix Pharmaceuticals Inc.) in accordance with instructions provided by the manufacturer. The adhesion to the protocol was evaluated by a nutritionist and failure to adhere resulted in the exclusion from the study.

### Statistical analysis

The results are presented as means ± SE. To compare mean values against those for controls, we employed one-way ANOVA with Dunnet’s least significant difference post-hoc tests. The 2-tailed Student’s *t* test was employed for 2-group comparisons. In correlation studies, the values of change in blood β-endorphin and *α*-MSH levels following a high-fat meal were obtained as the difference between post- and pre-meal values. The values obtained were used in correlation studies against the variation of body mass over a period of 15 days for humans or four weeks for rats. Thereafter, Pearson’s correlation coefficients were calculated, and univariate linear regression was used to test for significance. When necessary, analyses were complemented by the Tukey or Bonferroni tests to determine the significance of individual differences. The level of significance was set at p < 0.05.

## Additional Information

**How to cite this article**: Souza, G. F. P. *et al.* Defective regulation of POMC precedes hypothalamic inflammation in diet-induced obesity. *Sci. Rep.* **6**, 29290; doi: 10.1038/srep29290 (2016).

## Supplementary Material

Supplementary Information
