# Supplementary material for: Defective regulation of POMC precedes hypothalamic inflammation in diet-induced obesity
Source: Sci Rep. 2016 Jul 4;6:29290. doi: 10.1038/srep29290 (PMC4931679; doi:10.1038/srep29290)

Supplementary files

Defective regulation of POMC precedes hypothalamic inflammation in diet-induced obesity

Gabriela F. P. Souza[1], Carina Solon[1], Lucas F. Nascimento[1], Jose C. De-Lima-Junior[1], Guilherme Nogueira[1], Rodrigo Moura[1], Guilherme Z. Rocha[2], Milena Fioravante[1], Vanessa Bobbo[1,3], Joseane Morari[1], Daniela Razolli[1], Eliana P. Araujo[1,3], Licio A. Velloso[1,*]

[1]Laboratory of Cell Signaling, University of Campinas, 13084-970 – Campinas-SP, Brazil
[2]Department of Internal Medicine, University of Campinas, 13084-970 – Campinas-SP, Brazil
[3]Faculty of Nursing, University of Campinas, 13084-970 – Campinas-SP, Brazil
*Correspondence: lavelloso.unicamp@gmail.com

Supplementary Table 1. Anthropometric data of voluntaries.

Parameter	Value±SEM
Age (years)	22,1 ± 1,3
Heights (m)	1,78 ± 0,07
Initial Body Mass (Kg)	72,9 ± 9,7
Final Body Mass (Kg)	73,8 ± 10,3
Initial BMI (Kg/m^2)	23,1 ± 2,6
Final BMI (Kg/m^2)	23,7 ± 2,7
Initial Fat Mass (kg)	15,8 ± 5,0
Final Fat Mass (kg)	16,5 ± 5,1

Supplementary Table 2. Healthy diet recommendation. Voluntaries were instructed to consume *ad libitum*, for 5 days, foods present in this list, only.

Healthy Diet for 5 days
• Whole grains<br>• Whole breads<br>• Fruits and Vegetables<br>• Nuts<br>• Beans<br>• Fish<br>• Skimmed milk<br>• Yogurt<br>• Olive oil<br>• Lean meat<br>• Fruit juice

Supplementary Table 3. Fat-rich breakfast. Voluntaries were given foods present in this list to consume, *ad libitum*, during breakfast.

Fat-rich breakfast
• French bread<br>• Butter<br>• Cheese<br>• Bologna<br>• Industrialized chocolate milk drink<br>• Croissant stuffed with ham and cheese<br>• Industrialized cakes<br>• Cookies<br>• Candies

Supplementary Table 4. Fat-rich diet recommendation. Voluntaries were instructed to consume *ad libitum*, for 15 days, foods present in this list, only.

**Fat-Rich Diet for 15 days**
• Fat meats<br>• Sausages, salami, bologna, ham, bacon<br>• Hamburger<br>• Cheese bread<br>• Pizza<br>• Butter<br>• Fat cheese<br>• French fries<br>• Chocolate<br>• Cakes<br>• Candies<br>• Cookies<br>• Ice cream<br>• Fat milk<br>• Fast Food<br>• Fat industrialized food<br>• Deep-fried foods

Supplementary Figure 1. Protocol employed for the experiments shown in Supplementary Figure 2. Following determination of obesity resistance (OR) or obesity predisposition (OP), mice were submitted to an intracerebroventricular (icv) cannulation and treated with siRNA against POMC (in OP mice) or NPY (in OR mice). Scramble siRNA were employed as control. Thereafter, mice were fed on a HFD for one week and caloric intake and body mass were recorded.

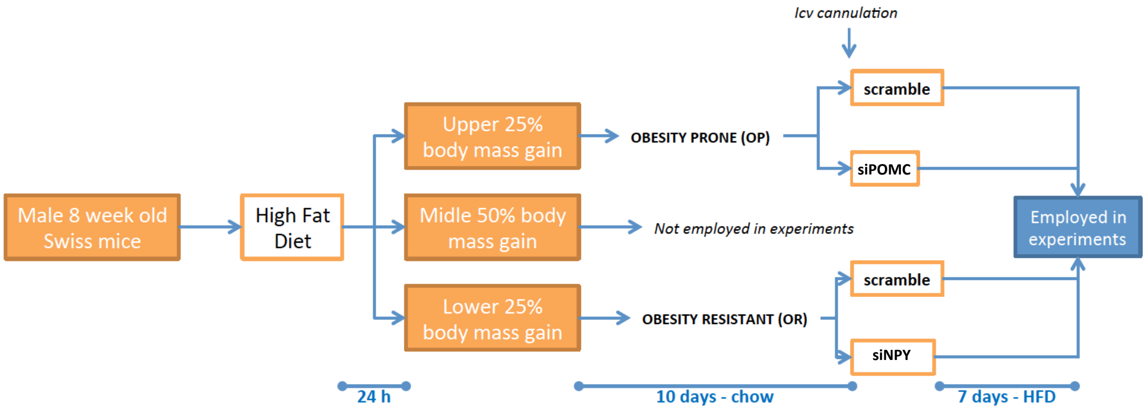

Supplementary Figure 2. Mice treated according to the protocol depicted in Supplementary Figure 1 were evaluated for food intake, which was record before beginning of the test (A) and after 24h (B), three days (C) or seven days on a high-fat diet. In addition, body mass variation was evaluated after three days (D) and seven days (E) on a high-fat diet. In all experiments n = 6. There were no significant intra-group (scramble vs. siRNA) differences.

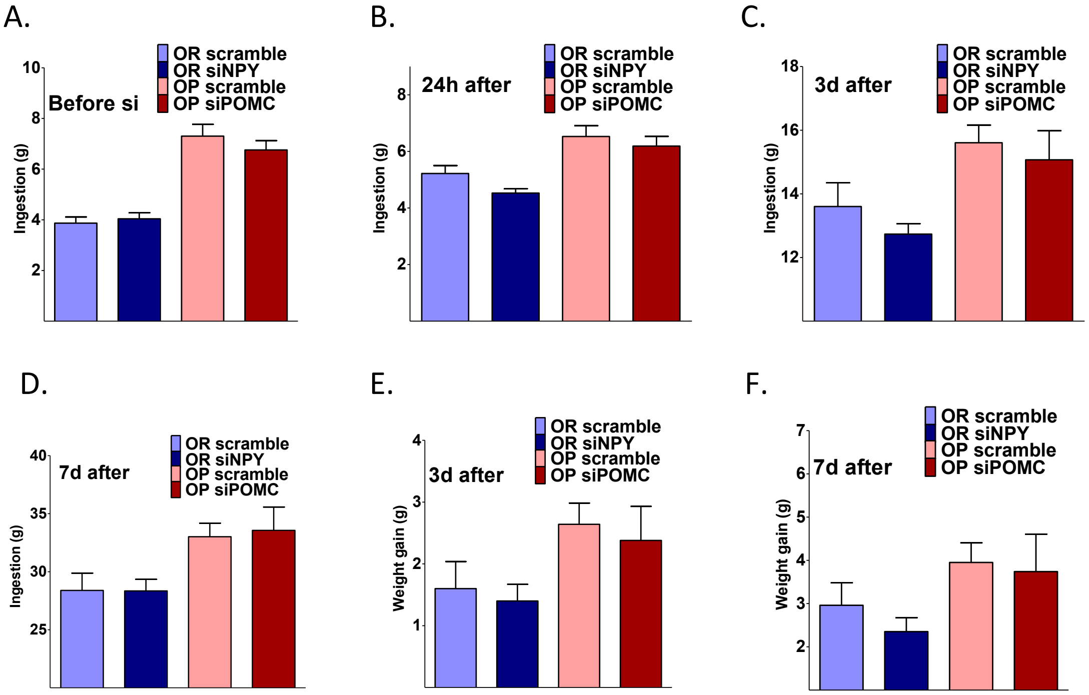

Supplement: Supplementary Information [file srep29290-s1.pdf]
